# Nanophotonics, nano-optics and nanospectroscopy

**DOI:** 10.3762/bjnano.2.53

**Published:** 2011-08-30

**Authors:** Alfred J Meixner

**Affiliations:** 1 Institute of Physical and Theoretical Chemistry, University of Tübingen, Auf der Morgenstelle 18, 72076 Tübingen, Germany

**Keywords:** nano-optics, nanophotonics, nanospectroscopy

This Thematic Series of the *Beilstein Journal of Nanotechnology* is devoted to nanophotonics, nano-optics and nanospectroscopy, and covers the field where nanoscience meets photonics, optics and spectroscopy. Since the pioneering days of scanning near-field optical microscopy [1–2], one of the main goals has been to combine scanning probe microscopy techniques with the spectroscopic means to characterize the chemical structure of materials with nanometer spatial resolution [3–6]. This has turned out to be a demanding but, at the same time, rewarding endeavor. The scaling down of optics and spectroscopy to the length scale of molecules is not simply a matter of making things smaller; the optical phenomena and spectroscopic behavior at the nanoscale are indeed markedly different from those at the macroscopic scale. This challenge continues to fascinate researchers all around the world and has led to many new discoveries concerning the interaction between light and matter at dimensions much smaller than the wavelength of electromagnetic radiation [7–9]. Well-known examples are the negative refractive index created by metamaterials [10–11], the quantum confinement observed in the absorption and luminescence spectra of semiconductor nanoparticles [12], and the plasmon resonances of silver and gold nanoparticles. The concept of plasmon resonance has led to broad applications, such as optical antennas made from noble metals, which have been used to locally focus light into volumes with dimensions far below the diffraction limit and to enhance the emission of locally excited states into the far field [13]. Equally important are fundamental studies of the optical properties of individual quantum systems, such as single atoms, single molecules [14] or single quantum dots, with high spectral resolution and high time resolution. A single molecule is the smallest chemical unit and can be regarded as a single-photon source; its optical properties demonstrate most naturally the quantum characteristics of light and reveal details of intermolecular interactions that would be otherwise hidden in an ensemble.

Nanophotonics and nanospectroscopy shine light into this intriguing new world. The study of the interaction between light and matter at the nanometer scale is motivated by the rapid progress in nanoscience and nanotechnology and requires the close cooperation of researchers from a number of different disciplines, including physics, chemistry and biology. Widespread applications can be imagined, e.g., in materials sciences in the pursuit of efficient photovoltaic energy conversion; in the engineering sciences as quantum devices functioning as switches that truly operate at the quantum limit with single photons; or in the life sciences as local optical sensors to observe chemical processes in living cells.

Tübingen, August 2011

Alfred J. Meixner
